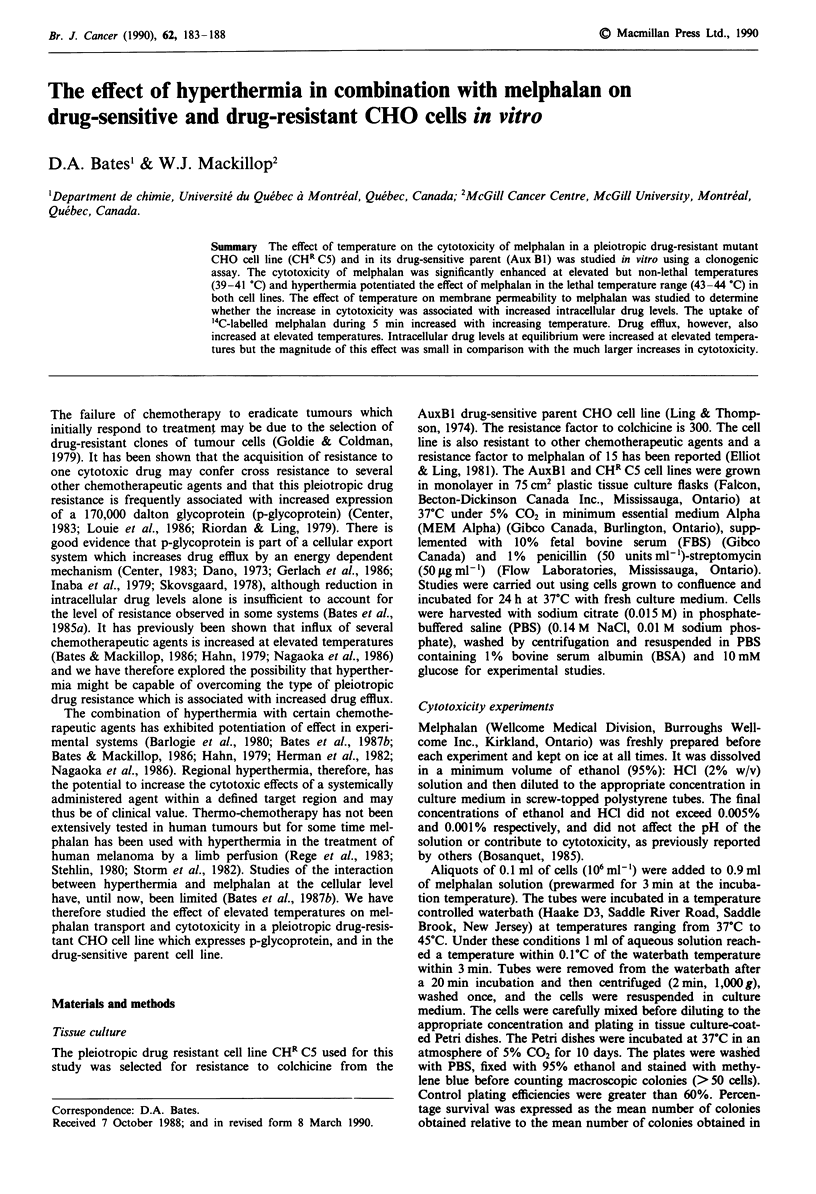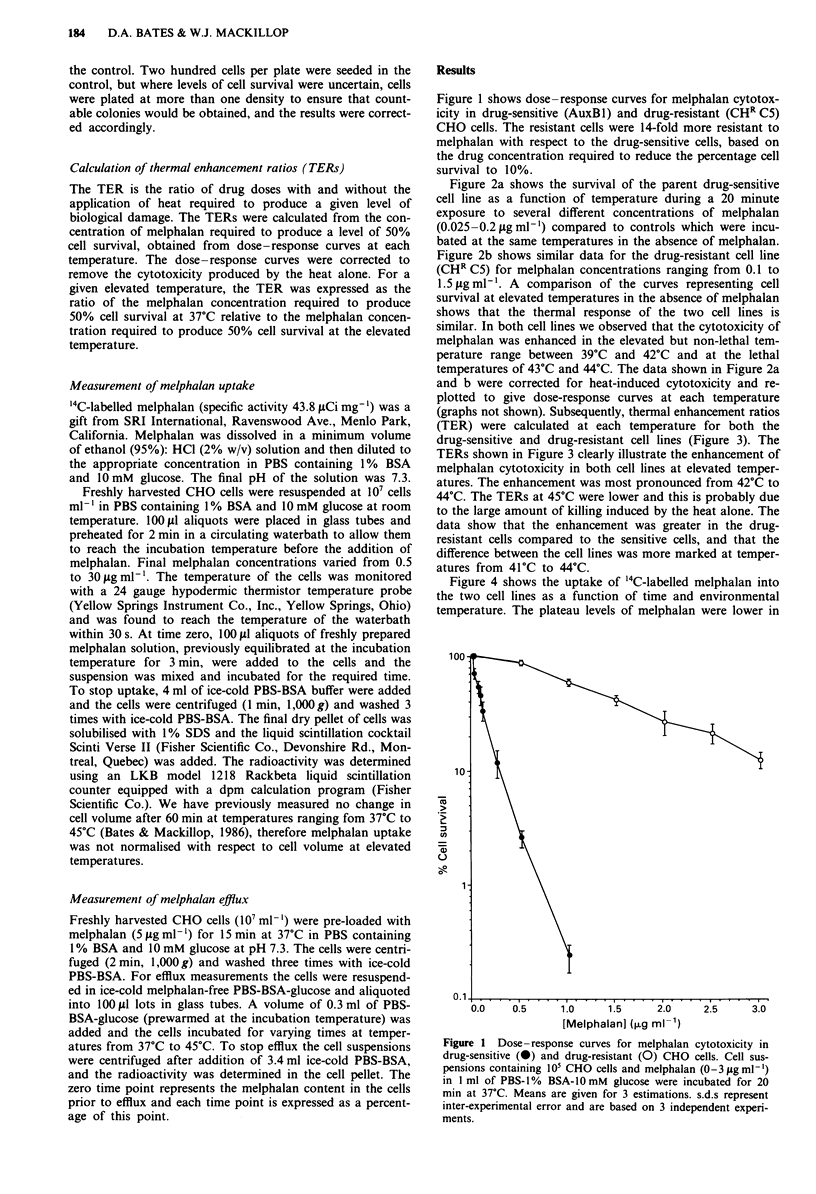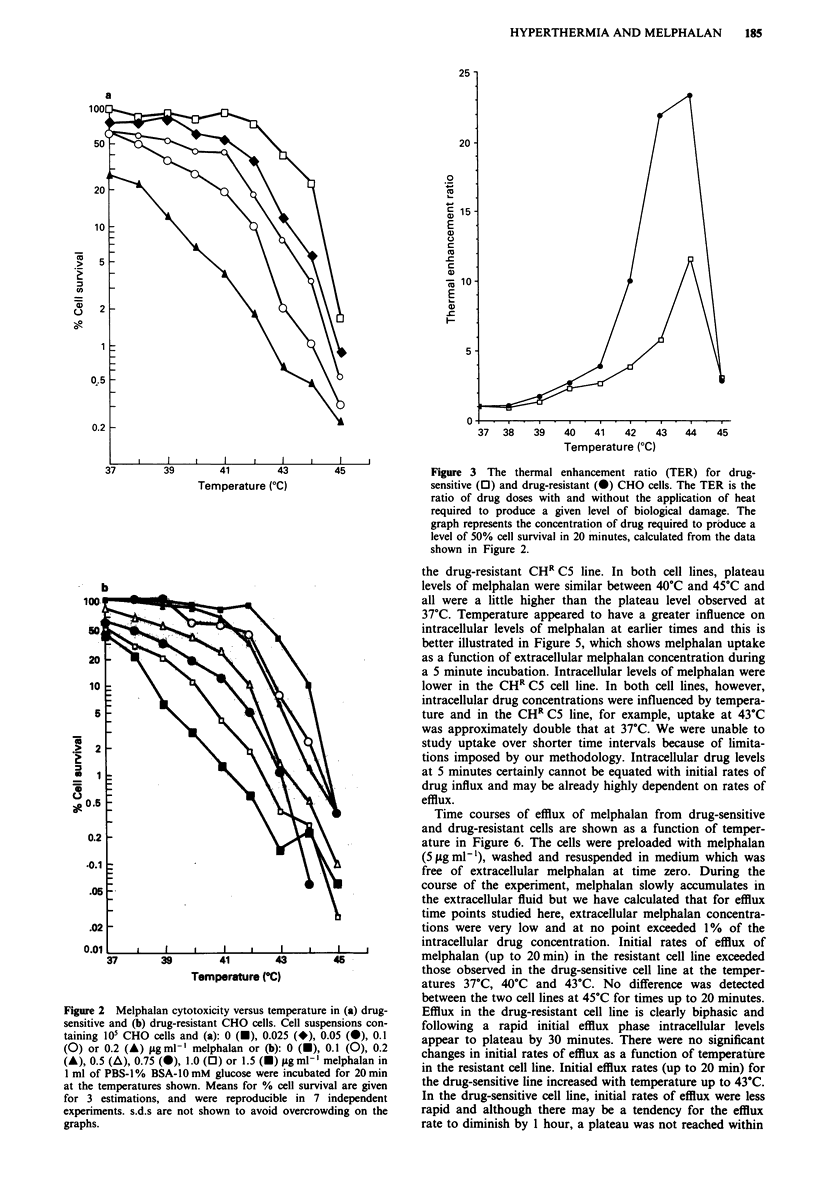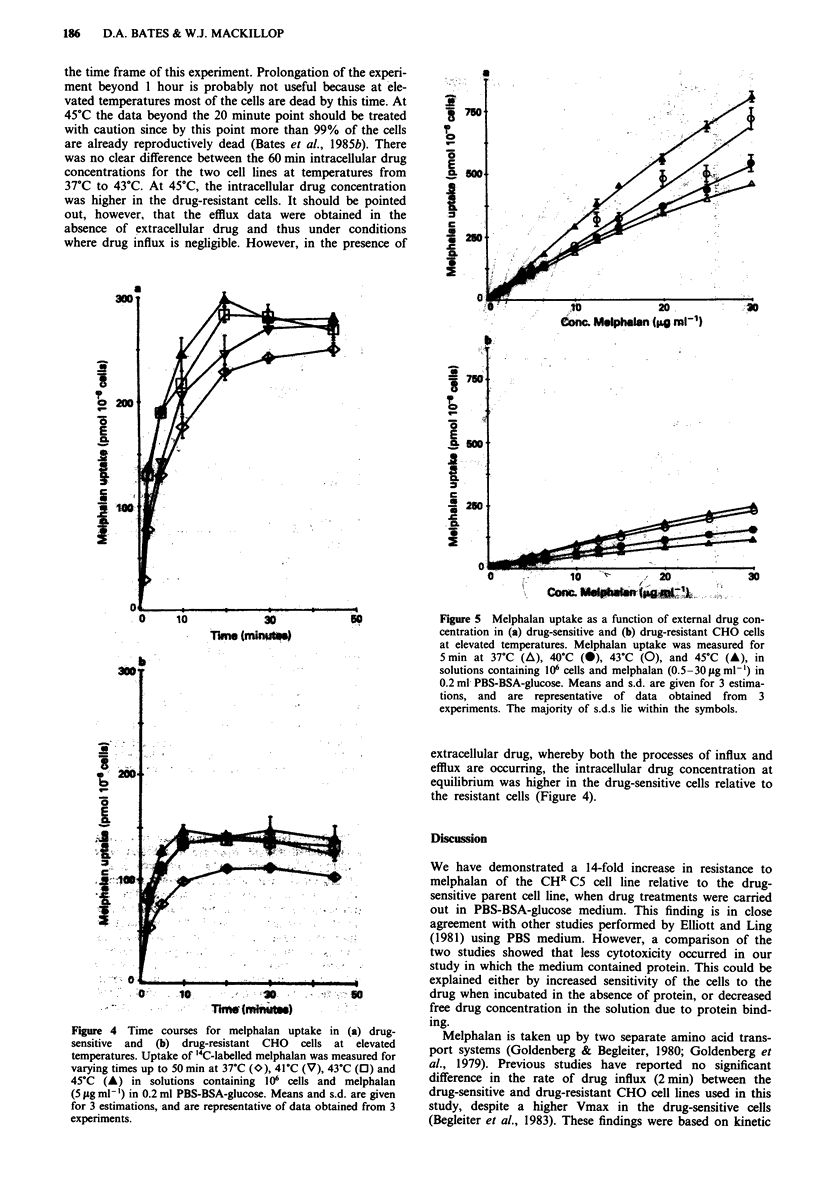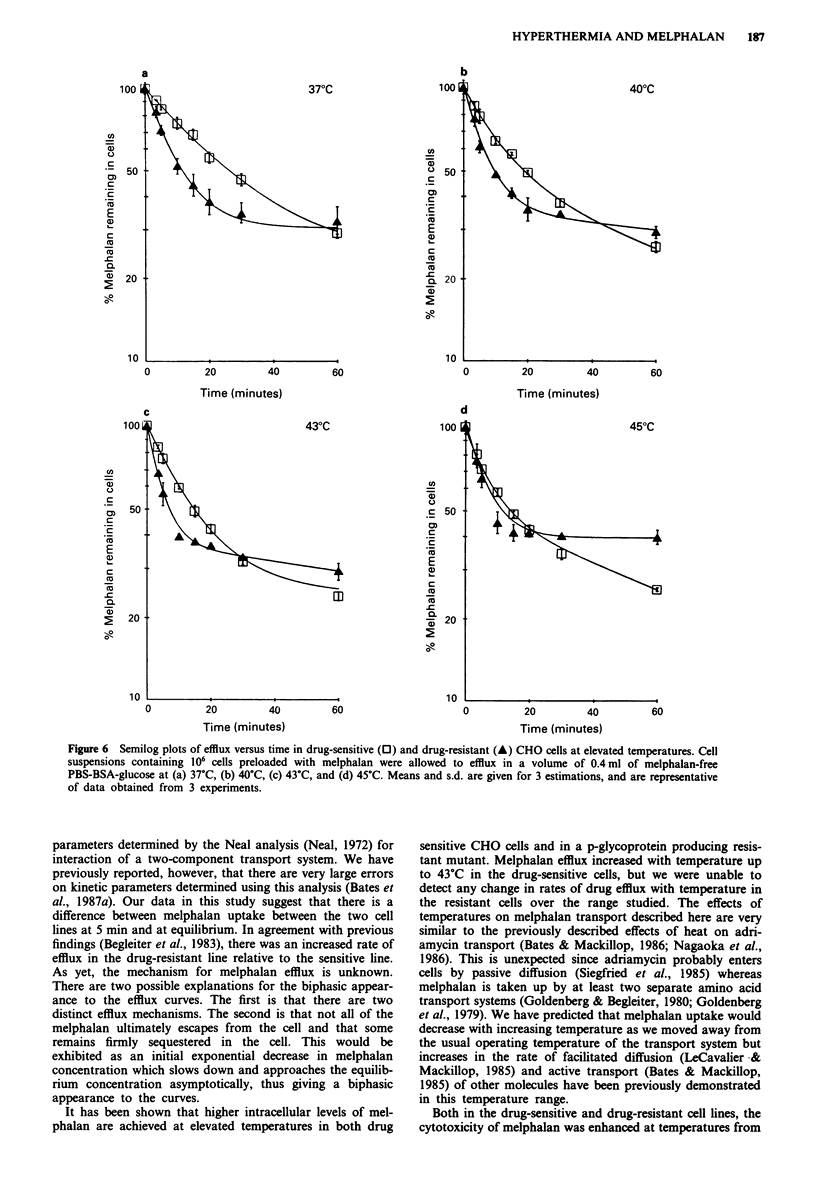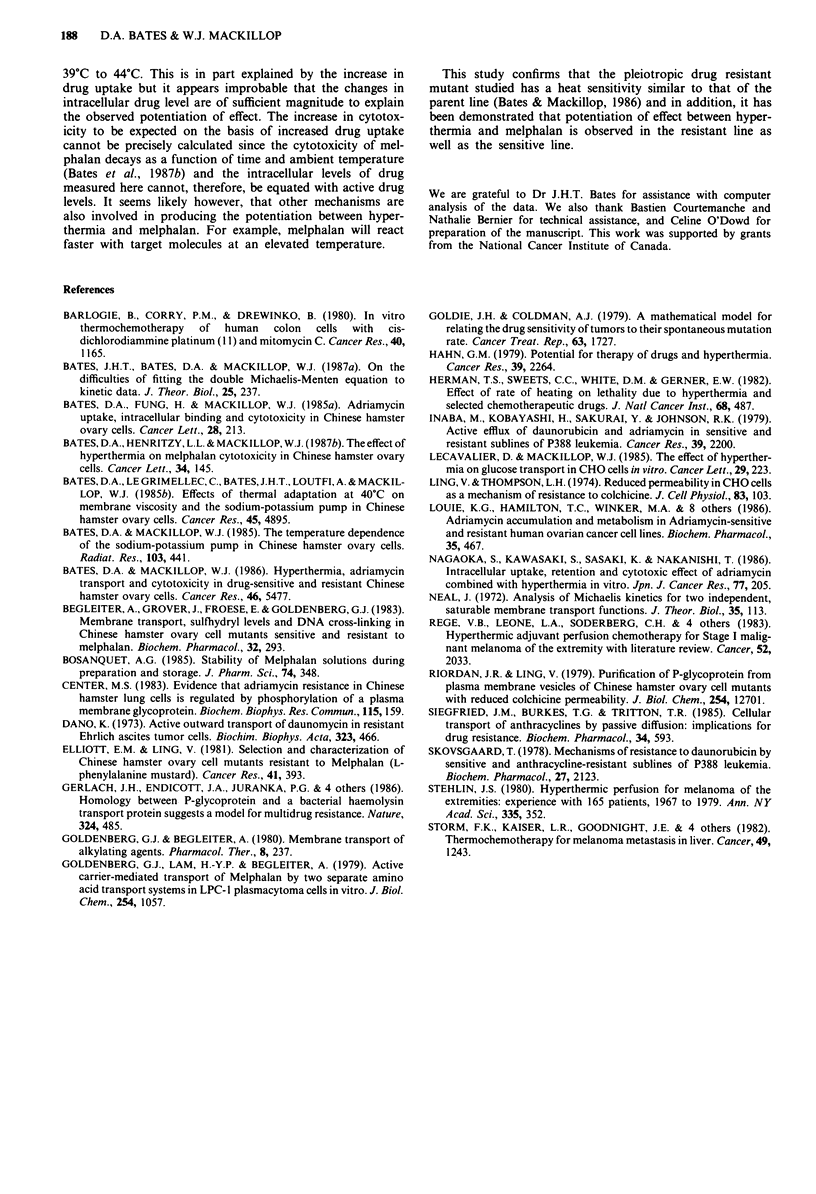# The effect of hyperthermia in combination with melphalan on drug-sensitive and drug-resistant CHO cells in vitro.

**DOI:** 10.1038/bjc.1990.257

**Published:** 1990-08

**Authors:** D. A. Bates, W. J. Mackillop

**Affiliations:** Department de Chimie, Université du Québec, Montréal, Canada.

## Abstract

The effect of temperature on the cytotoxicity of melphalan in a pleiotropic drug-resistant mutant CHO cell line (CHR C5) and in its drug-sensitive parent (Aux B1) was studied in vitro using a clonogenic assay. The cytotoxicity of melphalan was significantly enhanced at elevated but non-lethal temperatures (39-41 degrees C) and hyperthermia potentiated the effect of melphalan in the lethal temperature range (43-44 degrees C) in both cell lines. The effect of temperature on membrane permeability to melphalan was studied to determine whether the increase in cytotoxicity was associated with increased intracellular drug levels. The uptake of 14C-labelled melphalan during 5 min increased with increasing temperature. Drug efflux, however, also increased at elevated temperatures. Intracellular drug levels at equilibrium were increased at elevated temperatures but the magnitude of this effect was small in comparison with the much larger increases in cytotoxicity.


Br. J. Cancer (1990), 62, 183-188 © Macmillan Press Ltd., 1990

### The effect of hyperthermia in combination with melphalan on drug-sensitive and drug-resistant CHO cells *in vitro*

D.A. Bates[1] & W.J. Mackillop[2]

[1]*Department de chimie, Université du Québec à Montréal, Québec, Canada;* [2]*McGill Cancer Centre, McGill University, Montréal, Québec, Canada.*

**Summary** The effect of temperature on the cytotoxicity of melphalan in a pleiotropic drug-resistant mutant CHO cell line ($CH^R$ C5) and in its drug-sensitive parent (Aux B1) was studied *in vitro* using a clonogenic assay. The cytotoxicity of melphalan was significantly enhanced at elevated but non-lethal temperatures (39-41 °C) and hyperthermia potentiated the effect of melphalan in the lethal temperature range (43-44 °C) in both cell lines. The effect of temperature on membrane permeability to melphalan was studied to determine whether the increase in cytotoxicity was associated with increased intracellular drug levels. The uptake of $^{14}$C-labelled melphalan during 5 min increased with increasing temperature. Drug efflux, however, also increased at elevated temperatures. Intracellular drug levels at equilibrium were increased at elevated temperatures but the magnitude of this effect was small in comparison with the much larger increases in cytotoxicity.

The failure of chemotherapy to eradicate tumours which initially respond to treatment may be due to the selection of drug-resistant clones of tumour cells (Goldie & Coldman, 1979). It has been shown that the acquisition of resistance to one cytotoxic drug may confer cross resistance to several other chemotherapeutic agents and that this pleiotropic drug resistance is frequently associated with increased expression of a 170,000 dalton glycoprotein (p-glycoprotein) (Center, 1983; Louie *et al.*, 1986; Riordan & Ling, 1979). There is good evidence that p-glycoprotein is part of a cellular export system which increases drug efflux by an energy dependent mechanism (Center, 1983; Dano, 1973; Gerlach *et al.*, 1986; Inaba *et al.*, 1979; Skovsgaard, 1978), although reduction in intracellular drug levels alone is insufficient to account for the level of resistance observed in some systems (Bates *et al.*, 1985*a*). It has previously been shown that influx of several chemotherapeutic agents is increased at elevated temperatures (Bates & Mackillop, 1986; Hahn, 1979; Nagaoka *et al.*, 1986) and we have therefore explored the possibility that hyperthermia might be capable of overcoming the type of pleiotropic drug resistance which is associated with increased drug efflux.

The combination of hyperthermia with certain chemotherapeutic agents has exhibited potentiation of effect in experimental systems (Barlogie *et al.*, 1980; Bates *et al.*, 1987*b*; Bates & Mackillop, 1986; Hahn, 1979; Herman *et al.*, 1982; Nagaoka *et al.*, 1986). Regional hyperthermia, therefore, has the potential to increase the cytotoxic effects of a systemically administered agent within a defined target region and may thus be of clinical value. Thermo-chemotherapy has not been extensively tested in human tumours but for some time melphalan has been used with hyperthermia in the treatment of human melanoma by a limb perfusion (Rege *et al.*, 1983; Stehlin, 1980; Storm *et al.*, 1982). Studies of the interaction between hyperthermia and melphalan at the cellular level have, until now, been limited (Bates *et al.*, 1987*b*). We have therefore studied the effect of elevated temperatures on melphalan transport and cytotoxicity in a pleiotropic drug-resistant CHO cell line which expresses p-glycoprotein, and in the drug-sensitive parent cell line.

### Materials and methods

### *Tissue culture*

The pleiotropic drug resistant cell line $CH^R$ C5 used for this study was selected for resistance to colchicine from the AuxB1 drug-sensitive parent CHO cell line (Ling & Thompson, 1974). The resistance factor to colchicine is 300. The cell line is also resistant to other chemotherapeutic agents and a resistance factor to melphalan of 15 has been reported (Elliot & Ling, 1981). The AuxB1 and $CH^R$ C5 cell lines were grown in monolayer in 75 $cm^2$ plastic tissue culture flasks (Falcon, Becton-Dickinson Canada Inc., Mississauga, Ontario) at 37°C under 5% $CO_2$ in minimum essential medium Alpha (MEM Alpha) (Gibco Canada, Burlington, Ontario), supplemented with 10% fetal bovine serum (FBS) (Gibco Canada) and 1% penicillin (50 units $ml^{-1}$)-streptomycin (50 $\mu g\ ml^{-1}$) (Flow Laboratories, Mississauga, Ontario). Studies were carried out using cells grown to confluence and incubated for 24 h at 37°C with fresh culture medium. Cells were harvested with sodium citrate (0.015 M) in phosphate-buffered saline (PBS) (0.14 M NaCl, 0.01 M sodium phosphate), washed by centrifugation and resuspended in PBS containing 1% bovine serum albumin (BSA) and 10 mM glucose for experimental studies.

### *Cytotoxicity experiments*

Melphalan (Wellcome Medical Division, Burroughs Wellcome Inc., Kirkland, Ontario) was freshly prepared before each experiment and kept on ice at all times. It was dissolved in a minimum volume of ethanol (95%): HCl (2% w/v) solution and then diluted to the appropriate concentration in culture medium in screw-topped polystyrene tubes. The final concentrations of ethanol and HCl did not exceed 0.005% and 0.001% respectively, and did not affect the pH of the solution or contribute to cytotoxicity, as previously reported by others (Bosanquet, 1985).

Aliquots of 0.1 ml of cells ($10^6\ ml^{-1}$) were added to 0.9 ml of melphalan solution (prewarmed for 3 min at the incubation temperature). The tubes were incubated in a temperature controlled waterbath (Haake D3, Saddle River Road, Saddle Brook, New Jersey) at temperatures ranging from 37°C to 45°C. Under these conditions 1 ml of aqueous solution reached a temperature within 0.1°C of the waterbath temperature within 3 min. Tubes were removed from the waterbath after a 20 min incubation and then centrifuged (2 min, 1,000 *g*), washed once, and the cells were resuspended in culture medium. The cells were carefully mixed before diluting to the appropriate concentration and plating in tissue culture-coated Petri dishes. The Petri dishes were incubated at 37°C in an atmosphere of 5% $CO_2$ for 10 days. The plates were washed with PBS, fixed with 95% ethanol and stained with methylene blue before counting macroscopic colonies (> 50 cells). Control plating efficiencies were greater than 60%. Percentage survival was expressed as the mean number of colonies obtained relative to the mean number of colonies obtained in

Correspondence: D.A. Bates.
Received 7 October 1988; and in revised form 8 March 1990.

the control. Two hundred cells per plate were seeded in the control, but where levels of cell survival were uncertain, cells were plated at more than one density to ensure that countable colonies would be obtained, and the results were corrected accordingly.

*Calculation of thermal enhancement ratios (TERs)*

The TER is the ratio of drug doses with and without the application of heat required to produce a given level of biological damage. The TERs were calculated from the concentration of melphalan required to produce a level of 50% cell survival, obtained from dose–response curves at each temperature. The dose–response curves were corrected to remove the cytotoxicity produced by the heat alone. For a given elevated temperature, the TER was expressed as the ratio of the melphalan concentration required to produce 50% cell survival at 37°C relative to the melphalan concentration required to produce 50% cell survival at the elevated temperature.

*Measurement of melphalan uptake*

$^{14}$C-labelled melphalan (specific activity 43.8 $\mu$Ci mg$^{-1}$) was a gift from SRI International, Ravenswood Ave., Menlo Park, California. Melphalan was dissolved in a minimum volume of ethanol (95%): HCl (2% w/v) solution and then diluted to the appropriate concentration in PBS containing 1% BSA and 10 mM glucose. The final pH of the solution was 7.3.

Freshly harvested CHO cells were resuspended at $10^7$ cells ml$^{-1}$ in PBS containing 1% BSA and 10 mM glucose at room temperature. 100 $\mu$l aliquots were placed in glass tubes and preheated for 2 min in a circulating waterbath to allow them to reach the incubation temperature before the addition of melphalan. Final melphalan concentrations varied from 0.5 to 30 $\mu$g ml$^{-1}$. The temperature of the cells was monitored with a 24 gauge hypodermic thermistor temperature probe (Yellow Springs Instrument Co., Inc., Yellow Springs, Ohio) and was found to reach the temperature of the waterbath within 30 s. At time zero, 100 $\mu$l aliquots of freshly prepared melphalan solution, previously equilibrated at the incubation temperature for 3 min, were added to the cells and the suspension was mixed and incubated for the required time. To stop uptake, 4 ml of ice-cold PBS-BSA buffer were added and the cells were centrifuged (1 min, 1,000 *g*) and washed 3 times with ice-cold PBS-BSA. The final dry pellet of cells was solubilised with 1% SDS and the liquid scintillation cocktail Scinti Verse II (Fisher Scientific Co., Devonshire Rd., Montreal, Quebec) was added. The radioactivity was determined using an LKB model 1218 Rackbeta liquid scintillation counter equipped with a dpm calculation program (Fisher Scientific Co.). We have previously measured no change in cell volume after 60 min at temperatures ranging fom 37°C to 45°C (Bates & Mackillop, 1986), therefore melphalan uptake was not normalised with respect to cell volume at elevated temperatures.

*Measurement of melphalan efflux*

Freshly harvested CHO cells ($10^7$ ml$^{-1}$) were pre-loaded with melphalan (5 $\mu$g ml$^{-1}$) for 15 min at 37°C in PBS containing 1% BSA and 10 mM glucose at pH 7.3. The cells were centrifuged (2 min, 1,000 *g*) and washed three times with ice-cold PBS-BSA. For efflux measurements the cells were resuspended in ice-cold melphalan-free PBS-BSA-glucose and aliquoted into 100 $\mu$l lots in glass tubes. A volume of 0.3 ml of PBS-BSA-glucose (prewarmed at the incubation temperature) was added and the cells incubated for varying times at temperatures from 37°C to 45°C. To stop efflux the cell suspensions were centrifuged after addition of 3.4 ml ice-cold PBS-BSA, and the radioactivity was determined in the cell pellet. The zero time point represents the melphalan content in the cells prior to efflux and each time point is expressed as a percentage of this point.

### Results

Figure 1 shows dose–response curves for melphalan cytotoxicity in drug-sensitive (AuxB1) and drug-resistant (CH$^R$ C5) CHO cells. The resistant cells were 14-fold more resistant to melphalan with respect to the drug-sensitive cells, based on the drug concentration required to reduce the percentage cell survival to 10%.

Figure 2a shows the survival of the parent drug-sensitive cell line as a function of temperature during a 20 minute exposure to several different concentrations of melphalan (0.025–0.2 $\mu$g ml$^{-1}$) compared to controls which were incubated at the same temperatures in the absence of melphalan. Figure 2b shows similar data for the drug-resistant cell line (CH$^R$ C5) for melphalan concentrations ranging from 0.1 to 1.5 $\mu$g ml$^{-1}$. A comparison of the curves representing cell survival at elevated temperatures in the absence of melphalan shows that the thermal response of the two cell lines is similar. In both cell lines we observed that the cytotoxicity of melphalan was enhanced in the elevated but non-lethal temperature range between 39°C and 42°C and at the lethal temperatures of 43°C and 44°C. The data shown in Figure 2a and b were corrected for heat-induced cytotoxicity and replotted to give dose-response curves at each temperature (graphs not shown). Subsequently, thermal enhancement ratios (TER) were calculated at each temperature for both the drug-sensitive and drug-resistant cell lines (Figure 3). The TERs shown in Figure 3 clearly illustrate the enhancement of melphalan cytotoxicity in both cell lines at elevated temperatures. The enhancement was most pronounced from 42°C to 44°C. The TERs at 45°C were lower and this is probably due to the large amount of killing induced by the heat alone. The data show that the enhancement was greater in the drug-resistant cells compared to the sensitive cells, and that the difference between the cell lines was more marked at temperatures from 41°C to 44°C.

Figure 4 shows the uptake of $^{14}$C-labelled melphalan into the two cell lines as a function of time and environmental temperature. The plateau levels of melphalan were lower in

**Figure 1** Dose–response curves for melphalan cytotoxicity in drug-sensitive (●) and drug-resistant (○) CHO cells. Cell suspensions containing $10^5$ CHO cells and melphalan (0–3 $\mu$g ml$^{-1}$) in 1 ml of PBS-1% BSA-10 mM glucose were incubated for 20 min at 37°C. Means are given for 3 estimations. s.d.s represent inter-experimental error and are based on 3 independent experiments.

**Figure 2** Melphalan cytotoxicity versus temperature in (**a**) drug-sensitive and (**b**) drug-resistant CHO cells. Cell suspensions containing $10^5$ CHO cells and (**a**): 0 (■), 0.025 (◆), 0.05 (●), 0.1 (○) or 0.2 (▲) $\mu g\,ml^{-1}$ melphalan or (**b**): 0 (■), 0.1 (○), 0.2 (▲), 0.5 (△), 0.75 (●), 1.0 (□) or 1.5 (■) $\mu g\,ml^{-1}$ melphalan in 1 ml of PBS-1% BSA-10 mM glucose were incubated for 20 min at the temperatures shown. Means for % cell survival are given for 3 estimations, and were reproducible in 7 independent experiments. s.d.s are not shown to avoid overcrowding on the graphs.

**Figure 3** The thermal enhancement ratio (TER) for drug-sensitive (□) and drug-resistant (●) CHO cells. The TER is the ratio of drug doses with and without the application of heat required to produce a given level of biological damage. The graph represents the concentration of drug required to produce a level of 50% cell survival in 20 minutes, calculated from the data shown in Figure 2.

the drug-resistant $CH^R$ C5 line. In both cell lines, plateau levels of melphalan were similar between 40°C and 45°C and all were a little higher than the plateau level observed at 37°C. Temperature appeared to have a greater influence on intracellular levels of melphalan at earlier times and this is better illustrated in Figure 5, which shows melphalan uptake as a function of extracellular melphalan concentration during a 5 minute incubation. Intracellular levels of melphalan were lower in the $CH^R$ C5 cell line. In both cell lines, however, intracellular drug concentrations were influenced by temperature and in the $CH^R$ C5 line, for example, uptake at 43°C was approximately double that at 37°C. We were unable to study uptake over shorter time intervals because of limitations imposed by our methodology. Intracellular drug levels at 5 minutes certainly cannot be equated with initial rates of drug influx and may be already highly dependent on rates of efflux.

Time courses of efflux of melphalan from drug-sensitive and drug-resistant cells are shown as a function of temperature in Figure 6. The cells were preloaded with melphalan (5 $\mu g\,ml^{-1}$), washed and resuspended in medium which was free of extracellular melphalan at time zero. During the course of the experiment, melphalan slowly accumulates in the extracellular fluid but we have calculated that for efflux time points studied here, extracellular melphalan concentrations were very low and at no point exceeded 1% of the intracellular drug concentration. Initial rates of efflux of melphalan (up to 20 min) in the resistant cell line exceeded those observed in the drug-sensitive cell line at the temperatures 37°C, 40°C and 43°C. No difference was detected between the two cell lines at 45°C for times up to 20 minutes. Efflux in the drug-resistant cell line is clearly biphasic and following a rapid initial efflux phase intracellular levels appear to plateau by 30 minutes. There were no significant changes in initial rates of efflux as a function of temperature in the resistant cell line. Initial efflux rates (up to 20 min) for the drug-sensitive line increased with temperature up to 43°C. In the drug-sensitive cell line, initial rates of efflux were less rapid and although there may be a tendency for the efflux rate to diminish by 1 hour, a plateau was not reached within the time frame of this experiment. Prolongation of the experiment beyond 1 hour is probably not useful because at elevated temperatures most of the cells are dead by this time. At 45°C the data beyond the 20 minute point should be treated with caution since by this point more than 99% of the cells are already reproductively dead (Bates *et al.*, 1985*b*). There was no clear difference between the 60 min intracellular drug concentrations for the two cell lines at temperatures from 37°C to 43°C. At 45°C, the intracellular drug concentration was higher in the drug-resistant cells. It should be pointed out, however, that the efflux data were obtained in the absence of extracellular drug and thus under conditions where drug influx is negligible. However, in the presence of extracellular drug, whereby both the processes of influx and efflux are occurring, the intracellular drug concentration at equilibrium was higher in the drug-sensitive cells relative to the resistant cells (Figure 4).

**Figure 4** Time courses for melphalan uptake in (**a**) drug-sensitive and (**b**) drug-resistant CHO cells at elevated temperatures. Uptake of $^{14}$C-labelled melphalan was measured for varying times up to 50 min at 37°C (◇), 41°C (▽), 43°C (□) and 45°C (▲) in solutions containing $10^6$ cells and melphalan (5 μg ml$^{-1}$) in 0.2 ml PBS-BSA-glucose. Means and s.d. are given for 3 estimations, and are representative of data obtained from 3 experiments.

**Figure 5** Melphalan uptake as a function of external drug concentration in (**a**) drug-sensitive and (**b**) drug-resistant CHO cells at elevated temperatures. Melphalan uptake was measured for 5 min at 37°C (Δ), 40°C (●), 43°C (O), and 45°C (▲), in solutions containing $10^6$ cells and melphalan (0.5–30 μg ml$^{-1}$) in 0.2 ml PBS-BSA-glucose. Means and s.d. are given for 3 estimations, and are representative of data obtained from 3 experiments. The majority of s.d.s lie within the symbols.

### Discussion

We have demonstrated a 14-fold increase in resistance to melphalan of the CH$^R$ C5 cell line relative to the drug-sensitive parent cell line, when drug treatments were carried out in PBS-BSA-glucose medium. This finding is in close agreement with other studies performed by Elliott and Ling (1981) using PBS medium. However, a comparison of the two studies showed that less cytotoxicity occurred in our study in which the medium contained protein. This could be explained either by increased sensitivity of the cells to the drug when incubated in the absence of protein, or decreased free drug concentration in the solution due to protein binding.

Melphalan is taken up by two separate amino acid transport systems (Goldenberg & Begleiter, 1980; Goldenberg *et al.*, 1979). Previous studies have reported no significant difference in the rate of drug influx (2 min) between the drug-sensitive and drug-resistant CHO cell lines used in this study, despite a higher Vmax in the drug-sensitive cells (Begleiter *et al.*, 1983). These findings were based on kinetic

**Figure 6** Semilog plots of efflux versus time in drug-sensitive (□) and drug-resistant (▲) CHO cells at elevated temperatures. Cell suspensions containing $10^6$ cells preloaded with melphalan were allowed to efflux in a volume of 0.4 ml of melphalan-free PBS-BSA-glucose at (a) 37°C, (b) 40°C, (c) 43°C, and (d) 45°C. Means and s.d. are given for 3 estimations, and are representative of data obtained from 3 experiments.

parameters determined by the Neal analysis (Neal, 1972) for interaction of a two-component transport system. We have previously reported, however, that there are very large errors on kinetic parameters determined using this analysis (Bates *et al.*, 1987*a*). Our data in this study suggest that there is a difference between melphalan uptake between the two cell lines at 5 min and at equilibrium. In agreement with previous findings (Begleiter *et al.*, 1983), there was an increased rate of efflux in the drug-resistant line relative to the sensitive line. As yet, the mechanism for melphalan efflux is unknown. There are two possible explanations for the biphasic appearance to the efflux curves. The first is that there are two distinct efflux mechanisms. The second is that not all of the melphalan ultimately escapes from the cell and that some remains firmly sequestered in the cell. This would be exhibited as an initial exponential decrease in melphalan concentration which slows down and approaches the equilibrium concentration asymptotically, thus giving a biphasic appearance to the curves.

It has been shown that higher intracellular levels of melphalan are achieved at elevated temperatures in both drug sensitive CHO cells and in a p-glycoprotein producing resistant mutant. Melphalan efflux increased with temperature up to 43°C in the drug-sensitive cells, but we were unable to detect any change in rates of drug efflux with temperature in the resistant cells over the range studied. The effects of temperatures on melphalan transport described here are very similar to the previously described effects of heat on adriamycin transport (Bates & Mackillop, 1986; Nagaoka *et al.*, 1986). This is unexpected since adriamycin probably enters cells by passive diffusion (Siegfried *et al.*, 1985) whereas melphalan is taken up by at least two separate amino acid transport systems (Goldenberg & Begleiter, 1980; Goldenberg *et al.*, 1979). We have predicted that melphalan uptake would decrease with increasing temperature as we moved away from the usual operating temperature of the transport system but increases in the rate of facilitated diffusion (LeCavalier & Mackillop, 1985) and active transport (Bates & Mackillop, 1985) of other molecules have been previously demonstrated in this temperature range.

Both in the drug-sensitive and drug-resistant cell lines, the cytotoxicity of melphalan was enhanced at temperatures from

39°C to 44°C. This is in part explained by the increase in drug uptake but it appears improbable that the changes in intracellular drug level are of sufficient magnitude to explain the observed potentiation of effect. The increase in cytotoxicity to be expected on the basis of increased drug uptake cannot be precisely calculated since the cytotoxicity of melphalan decays as a function of time and ambient temperature (Bates *et al.*, 1987*b*) and the intracellular levels of drug measured here cannot, therefore, be equated with active drug levels. It seems likely however, that other mechanisms are also involved in producing the potentiation between hyperthermia and melphalan. For example, melphalan will react faster with target molecules at an elevated temperature.

This study confirms that the pleiotropic drug resistant mutant studied has a heat sensitivity similar to that of the parent line (Bates & Mackillop, 1986) and in addition, it has been demonstrated that potentiation of effect between hyperthermia and melphalan is observed in the resistant line as well as the sensitive line.

We are grateful to Dr J.H.T. Bates for assistance with computer analysis of the data. We also thank Bastien Courtemanche and Nathalie Bernier for technical assistance, and Celine O'Dowd for preparation of the manuscript. This work was supported by grants from the National Cancer Institute of Canada.

### References

BARLOGIE, B., CORRY, P.M., & DREWINKO, B. (1980). In vitro thermochemotherapy of human colon cells with cis-dichlorodiammine platinum (11) and mitomycin C. *Cancer Res.*, **40**, 1165.

BATES, J.H.T., BATES, D.A. & MACKILLOP, W.J. (1987*a*). On the difficulties of fitting the double Michaelis-Menten equation to kinetic data. *J. Theor. Biol.*, **25**, 237.

BATES, D.A., FUNG, H. & MACKILLOP, W.J. (1985*a*). Adriamycin uptake, intracellular binding and cytotoxicity in Chinese hamster ovary cells. *Cancer Lett.*, **28**, 213.

BATES, D.A., HENRITZY, L.L. & MACKILLOP, W.J. (1987*b*). The effect of hyperthermia on melphalan cytotoxicity in Chinese hamster ovary cells. *Cancer Lett.*, **34**, 145.

BATES, D.A., LE GRIMELLEC, C., BATES, J.H.T., LOUTFI, A. & MACKILLOP, W.J. (1985*b*). Effects of thermal adaptation at 40°C on membrane viscosity and the sodium-potassium pump in Chinese hamster ovary cells. *Cancer Res.*, **45**, 4895.

BATES, D.A. & MACKILLOP, W.J. (1985). The temperature dependence of the sodium-potassium pump in Chinese hamster ovary cells. *Radiat. Res.*, **103**, 441.

BATES, D.A. & MACKILLOP, W.J. (1986). Hyperthermia, adriamycin transport and cytotoxicity in drug-sensitive and resistant Chinese hamster ovary cells. *Cancer Res.*, **46**, 5477.

BEGLEITER, A., GROVER, J., FROESE, E. & GOLDENBERG, G.J. (1983). Membrane transport, sulfhydryl levels and DNA cross-linking in Chinese hamster ovary cell mutants sensitive and resistant to melphalan. *Biochem. Pharmacol.*, **32**, 293.

BOSANQUET, A.G. (1985). Stability of Melphalan solutions during preparation and storage. *J. Pharm. Sci.*, **74**, 348.

CENTER, M.S. (1983). Evidence that adriamycin resistance in Chinese hamster lung cells is regulated by phosphorylation of a plasma membrane glycoprotein. *Biochem. Biophys. Res. Commun.*, **115**, 159.

DANO, K. (1973). Active outward transport of daunomycin in resistant Ehrlich ascites tumor cells. *Biochim. Biophys. Acta*, **323**, 466.

ELLIOTT, E.M. & LING, V. (1981). Selection and characterization of Chinese hamster ovary cell mutants resistant to Melphalan (L-phenylalanine mustard). *Cancer Res.*, **41**, 393.

GERLACH, J.H., ENDICOTT, J.A., JURANKA, P.G. & 4 others (1986). Homology between P-glycoprotein and a bacterial haemolysin transport protein suggests a model for multidrug resistance. *Nature*, **324**, 485.

GOLDENBERG, G.J. & BEGLEITER, A. (1980). Membrane transport of alkylating agents. *Pharmacol. Ther.*, **8**, 237.

GOLDENBERG, G.J., LAM, H.-Y.P. & BEGLEITER, A. (1979). Active carrier-mediated transport of Melphalan by two separate amino acid transport systems in LPC-1 plasmacytoma cells in vitro. *J. Biol. Chem.*, **254**, 1057.

GOLDIE, J.H. & COLDMAN, A.J. (1979). A mathematical model for relating the drug sensitivity of tumors to their spontaneous mutation rate. *Cancer Treat. Rep.*, **63**, 1727.

HAHN, G.M. (1979). Potential for therapy of drugs and hyperthermia. *Cancer Res.*, **39**, 2264.

HERMAN, T.S., SWEETS, C.C., WHITE, D.M. & GERNER, E.W. (1982). Effect of rate of heating on lethality due to hyperthermia and selected chemotherapeutic drugs. *J. Natl Cancer Inst.*, **68**, 487.

INABA, M., KOBAYASHI, H., SAKURAI, Y. & JOHNSON, R.K. (1979). Active efflux of daunorubicin and adriamycin in sensitive and resistant sublines of P388 leukemia. *Cancer Res.*, **39**, 2200.

LECAVALIER, D. & MACKILLOP, W.J. (1985). The effect of hyperthermia on glucose transport in CHO cells *in vitro*. *Cancer Lett.*, **29**, 223.

LING, V. & THOMPSON, L.H. (1974). Reduced permeability in CHO cells as a mechanism of resistance to colchicine. *J. Cell Physiol.*, **83**, 103.

LOUIE, K.G., HAMILTON, T.C., WINKER, M.A. & 8 others (1986). Adriamycin accumulation and metabolism in Adriamycin-sensitive and resistant human ovarian cancer cell lines. *Biochem. Pharmacol.*, **35**, 467.

NAGAOKA, S., KAWASAKI, S., SASAKI, K. & NAKANISHI, T. (1986). Intracellular uptake, retention and cytotoxic effect of adriamycin combined with hyperthermia in vitro. *Jpn. J. Cancer Res.*, **77**, 205.

NEAL, J. (1972). Analysis of Michaelis kinetics for two independent, saturable membrane transport functions. *J. Theor. Biol.*, **35**, 113.

REGE, V.B., LEONE, L.A., SODERBERG, C.H. & 4 others (1983). Hyperthermic adjuvant perfusion chemotherapy for Stage I malignant melanoma of the extremity with literature review. *Cancer*, **52**, 2033.

RIORDAN, J.R. & LING, V. (1979). Purification of P-glycoprotein from plasma membrane vesicles of Chinese hamster ovary cell mutants with reduced colchicine permeability. *J. Biol. Chem.*, **254**, 12701.

SIEGFRIED, J.M., BURKES, T.G. & TRITTON, T.R. (1985). Cellular transport of anthracyclines by passive diffusion: implications for drug resistance. *Biochem. Pharmacol.*, **34**, 593.

SKOVSGAARD, T. (1978). Mechanisms of resistance to daunorubicin by sensitive and anthracycline-resistant sublines of P388 leukemia. *Biochem. Pharmacol.*, **27**, 2123.

STEHLIN, J.S. (1980). Hyperthermic perfusion for melanoma of the extremities: experience with 165 patients, 1967 to 1979. *Ann. NY Acad. Sci.*, **335**, 352.

STORM, F.K., KAISER, L.R., GOODNIGHT, J.E. & 4 others (1982). Thermochemotherapy for melanoma metastasis in liver. *Cancer*, **49**, 1243.